# SPECT/CT Scan Images to Evaluate COVID-19 Pulmonary Complications: A Systematic Review

**DOI:** 10.3390/ijerph22020308

**Published:** 2025-02-18

**Authors:** Ana Carolina Coelho-Oliveira, Redha Taiar, Luelia Teles Jaques-Albuquerque, Ana Gabriellie Valério-Penha, Aline Reis-Silva, Luiz Felipe Ferreira-Souza, Danúbia da Cunha de Sá-Caputo, Mario Bernardo-Filho

**Affiliations:** 1 Programa de Pós-Graduação em Fisiopatologia Clínica e Experimental, Universidade do Estado do Rio de Janeiro, Rio de Janeiro 20551-030, RJ, Brazil; anagabriellie.vpenha@gmail.com (A.G.V.-P.); dradanubia@gmail.com (D.d.C.d.S.-C.); 2 Laboratório de Vibrações Mecânicas e Práticas Integrativas, Instituto de Biologia Roberto Alcantara Gomes, Universidade do Estado do Rio de Janeiro, Rio de Janeiro 20950-003, RJ, Brazil; lueliaa19@gmail.com (L.T.J.-A.); fisio.alinereis@hotmail.com (A.R.-S.); lumadaragu@gmail.com (L.F.F.-S.); bernardofilhom@gmail.com (M.B.-F.); 3 Université de Reims, MATériaux et Ingénierie Mécanique (MATIM), 51687 Reims Cedex 2, France; redha.taiar@univ-reims.fr; 4 Programa de Pós-Graduação em Ciências Médicas, Faculdade de Ciências Médicas, Universidade do Estado do Rio de Janeiro, Rio de Janeiro 20551-170, RJ, Brazil

**Keywords:** SPECT images, single positron emission tomography, coronavirus, lung abnormalities, lung perfusion

## Abstract

Introduction: The highly contagious 2019 novel coronavirus that causes coronavirus disease 2019 increased the scientific community’s interest in diagnosing and monitoring COVID-19. Due to the findings about the association between COVID-19 infection and pulmonary disturbances, the need for the use of complementary tests that can be carried out, preserving the health of patients, has grown. In this context, single-photon emission computed tomography (SPECT) was performed during the COVID-19 pandemic to assess and try to diagnose lung lesions. The aim of this current review was to investigate the types of SPECT images most commonly used and the main pulmonary parenchymal lesions and different lung perfusion abnormalities observed in these images in individuals with COVID-19 in different countries in the world. Materials and Methods: Electronic searches in the MEDLINE/PubMed, Embase, Scopus, Web of Science, and CINAHL databases were conducted in December 2022. Studies that used SPECT/CT scans to evaluate pulmonary involvements due to COVID-19, with no language restriction, were included. Two reviewers, who independently examined titles and abstracts, identified records through the database search and reference screening, and irrelevant studies were excluded based on the eligibility criteria. Relevant complete texts were analyzed for eligibility, and all relevant studies were included in a systematic review. Results: Eight studies with regular methodological quality were included. The types of SPECT examinations used in the included articles were SPECT/CT, Q SPECT/CT, and V/Q SPECT. The possible pulmonary complication most observed was pulmonary embolism. Conclusions: This systematic review demonstrated that SPECT/CT scans, mainly with perfusion methods, allow the maximum extraction of benefits from pulmonary images, in safety, suggesting efficiency in the differential diagnosis, including of respiratory diseases of different etiology, and with diagnostics and additional analyses, can possibly aid the development of suitable therapeutic strategies for each patient. Randomized clinical trials and studies of good methodological quality are necessary to confirm the findings of this review and help better understand the types of SPECT images most commonly used and the main pulmonary parenchymal lesions observed in the images in individuals with COVID-19.

## 1. Introduction

Coronavirus disease 2019 (COVID-19), known as severe acute respiratory syndrome coronavirus 2 (SARS-CoV-2), turned into one of the biggest challenges in the recent history of medicine [1]. The lung is the target organ, but the clinical manifestations of this disease are variable and range from incidental findings in an asymptomatic patient to acute respiratory injury, which can cause respiratory sequelae [2]. Computed tomography (CT) was frequently performed during the COVID-19 pandemic to assess lung lesions as well as to evaluate lesions with ground-glass opacities (GGOs), crazy-paving (CP) lesions, and pulmonary consolidations or to look for multiple complications, such as pulmonary embolism (PE) or pulmonary thromboembolism, pulmonary infiltrates from COVID pneumonia, and perfusion shunting [2].

Therefore, in patients with COVID-19, imaging follow-up is essential to observe and identify pulmonary abnormalities, and it is also important for assessing the effect of therapy, differentiating conditions, and helping to explain physical disabilities [3].

Among the possible exams, computed tomography pulmonary angiography (CTPA) is an imaging alternative for pulmonary arteries, for example, for the diagnosis of acute PE, but contraindications to intravenous contrast material (e.g., an allergy to iodine-based contrast agents or severe renal failure) may preclude patients from undergoing the examination [4]. In this case, nuclear medicine exams are desirable, such as planar ventilation/perfusion scans (V/Q scans) and ventilation/perfusion single-photon emission computed tomography (V/Q SPECT), either combined with low-dose computed tomography (ldCT) or perfusion (Q) only with SPECT with CT (Q-only SPECT/CT), which may be examination and diagnostics alternatives for patients with COVID-19 that also give information on pulmonary structural abnormalities [5,6].

In this sense, SPECT images are important because they allow a functional and metabolic assessment of organs and tissues, unlike anatomical images obtained by radiography, CT, or magnetic resonance imaging. In the pulmonary context, within the scope of COVID-19, SPECT images are widely used to detect and provide functional diagnosis, including diagnosing PE and assessing pulmonary perfusion and ventilation, in addition to being useful in staging lung diseases and evaluating pulmonary complications related to SARS-CoV-2 infection [7].

However, in patients with positive COVID-19, due to the potential for the secretion of respiratory droplets containing viruses, a ventilation scan is recognized as a procedure that requires caution. It has been suggested that ventilation can be replaced by perfusion-only SPECT combined with CT to minimize the potential exposure to aerosolized secretions and respiratory droplets in the nuclear medicine department [1,4]. However, this type of exam also ends up being performed in individuals with COVID-19.

From a review of the literature, few studies of good methodological quality have examined COVID-19 patient populations using scintigraphy, and there is only one review that addresses different scintigraphy protocols in the COVID-19 era [3]. However, so far, no research is available that describes the abnormalities seen on different SPECT/CT scans in a population suffering from active COVID-19. Therefore, the aim of the current study was to investigate the types of SPECT images most commonly used and the main pulmonary parenchymal lesions and different lung perfusion abnormalities observed in these images in individuals with COVID-19 in different countries in the world.

## 2. Materials and Methods

The methods for this review were based on the Preferred Reporting Items for Systematic Reviews and Meta-Analysis (PRISMA) guidelines [8], and the protocol was registered with the International Prospective Register of Systematic Reviews—PROSPERO (number CRD42022371301).

Search strategy: The search string was applied to appendices involving the following descriptors: ((COVID 19 OR COVID-19 OR coronavirus) AND lung AND (spect OR “CT Scan, Single Photon Emission” OR “Single-Photon Emission Computerized Tomography”)). The electronic search was conducted in the PubMed, Embase, Scopus, Web of Science, and CINAHL databases on 22 December 2022. The keywords used in the search were defined based on the PICO strategy [9] and focused on patients with COVID-19 (Participants), studies using SPECT/CT scans, perfusion SPECT CT (Q SPECT/CT), or ventilation/perfusion SPECT CT (V/Q SPECT/CT) for the diagnosis of pulmonary complications (Intervention), with comparisons between SPECT image types (Comparison). All reported outcomes regarding SPECT image scans to identify pulmonary complications were considered if deemed relevant to the studied topic and population (Outcomes). Additionally, a hand search was performed of the gray literature and included study references. This strategy allowed us to consider all publications relevant to the studied population, and it allowed us to answer the question “What is the current evidence on the types of SPECT scan images and the pulmonary parenchymal lesions identified in individuals with COVID-19?”.

**Figure 1:**
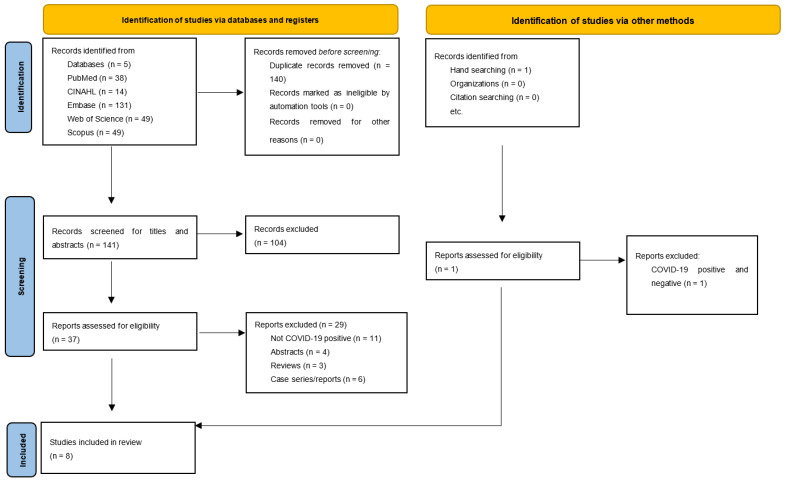
PRISMA 2020 flowchart of the selected studies. (From: Page MJ, McKenzie JE, Bossuyt PM, Boutron I, Hoffmann TC, Mulrow CD, et al. The PRISMA 2020 statement: an updated guideline for reporting systematic reviews. BMJ 2021;372:n71. doi: 10.1136/bmj.n71. [8]).

Eligibility criteria: Inclusion criteria: To be included in this review, publications had to meet the search criteria and had to investigate the use of only SPECT, Q SPECT, or V/Q SPECT scans to assess the complications of COVID-19; be related to individuals with a positive COVID-19 diagnosis; be complete articles, a retrospective study, or a short communication; have no language restrictions; and be independent of the year of publication. Exclusion criteria: Papers were excluded if they (i) had findings not related to some kind of SPECT image and COVID-19; (ii) were case series/reports, replies, editorials, letters, abstracts, reviews, or books; and (iii) had findings related to SPECT and other types of images different to SPECT. A flowchart (Figure 1), based in the PRISMA analysis, shows the steps in the selection of the complete papers analyzed in this review [8].

Study selection and data extraction: After searching all the databases, all duplicates were removed by the Endnote X9 software. Then, two independent reviewers analyzed the titles and abstracts, and irrelevant studies were excluded based on the eligibility criteria. For the studies considered relevant, the complete texts were read, and the studies that fit the criteria were selected. The same reviewers were responsible for extracting the data. Disagreements between reviewers at any stage of the review were resolved by a third reviewer. Data regarding study information (author and year), study design, demographics (sample size and age), the severity of COVID-19, the SPECT exam type, the radiopharmaceutical used, lung complications, diagnoses (pre- and post-exam), and the results were extracted.

Quality assessment and risk of bias: The evaluation of the risk of bias and the quality assessment of the selected studies was conducted using the Cochrane ROBINS-I software, a tool for assessing the risk of bias in non-randomized studies of interventions. ROBINS-I contains questions to guide risk-of-bias judgments within seven bias domains to compare the health effects of interventions (pre-intervention, during the intervention, and post-intervention), and each topic is classified as having a low, moderate, serious, or critical risk of bias and is required even when no information is present [10].

Level of evidence: The National Health and Medical Research Council hierarchy of evidence (NHMRC) was assessed in each study by the level of evidence of each selected publication, defined as follows: I—systematic review of randomized studies; II—randomized controlled trial (RCT); III-1—pseudo-randomized controlled trial; III-2—comparative study with concurrent controls (non-randomized experimental trial); III-3—comparative study without concurrent control; IV—case series with either post-test or pre-test/post-test outcomes [11].

## 3. Results

A total of 281 studies were identified in the databases used (PubMed: 38; CINAHL: 14; Embase: 131; Web of Science: 49; Scopus: 49), and after removing 140 duplicates, 141 articles were identified. During the evaluation process, 104 articles were excluded for not discussing the topic in question, for being a case series or case report, for being review or editorial articles, and for cardiac SPECT/CT images, positron emission tomography (PET) scans, or SPECT scans. The remaining 37 eligible studies were separated out to be read in full, and after a detailed reading, were excluded if they were, for example, a review, an abstract, a case series/report, or not related to individuals who were COVID-19 positive. Finally, eight studies were selected for this systematic review, as can be seen in Figure 1.

According to Table 1, of the eight studies included in this review, two were considered a single-center retrospective study [12,13], one a retrospective analysis [14], one a multicenter study [15], one a retrospective cohort study [16], one a retrospective cross-sectional study [17], and one a retrospective short communication [18]; one study [19] did not provide information about the type of study. Nevertheless, despite the different study designs, with respect to the level of evidence, all the included studies were considered level III-2.

All patients in the included studies had a confirmed diagnosis of infection with COVID-19, and at the time of the study, there were hospitalized and non-hospitalized patients.

The type of scan performed was SPECT/CT in five studies [13,14,15,16], Q SPECT/CT in three studies [12,17,18], and V/Q SPECT in three studies [14,15,16]. The radiopharmaceutical activity varied in terms of perfusion technique protocols, using 3–5 mCi of 99 mTc MAA [16,17] or 4–6 mCi of 99 mTc MAA [13], and one study did not specify the radiopharmaceutical used, which was identified only as a dose of 1–1.5 MBq per kg [19]. Additionally, one study had a perfusion protocol but did not use contrast [18], and in the case of ventilation techniques, the radiopharmaceuticals were 99 mTc-Technegas^®^ and/or krypton gas (81 mKr) [14,15] or 20–25 mCi of 99 mTc-DTPA [16].

In general, only one study [19] did not observe the presence of PE as a pulmonary complication in the participants, but interstitial lung diseases (pulmonary fibrosis, bronchiectasis) were observed. Increased D-dimer levels were found in four studies [13,16,17,18], and the other studies [12,14,15,19] did not assess D-dimer levels. The sample size described in the publications ranged from 6 to 183 participants.

**Figure 2:**
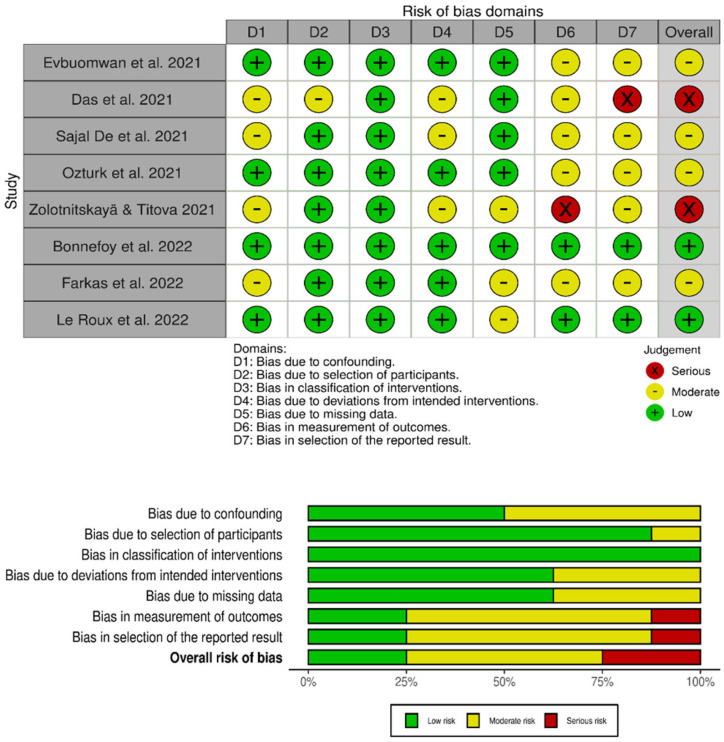
Quality assessment for the selected studies [12,13,14,15,16,17,18,19].

Figure 2 shows the results in relation to the overall judgment of risk of bias, and after analysis based on the ROBINS-I tool, the final classification was low RoB for two studies due to loss of data [18,19], moderate RoB for four studies due to flaws mainly regarding the measurement of the results and the selection of the reported results [12,13,16,17], and two studies with severe RoB that had important methodological flaws regarding the measurement of the results and the selection of the reported results [14,15].

## 4. Discussion

As noted in the results of the current review, coronavirus can spread from person to person very quickly through respiratory droplets, which is one of the main reasons for the global pandemic, and in this context, currently, although ventilation tests are still performed, perfusion scans through Q-SPECT/CT have higher safety and accuracy than ventilation/perfusion scintigraphy for the diagnostics of pulmonary abnormalities.

With regard to pulmonary abnormalities, the presence of PE was nearly unanimous in the included studies [12,13,14,15,16,17,18], not being observed in only one study [19] where other microcirculation disorders were identified. The overlapping of signs and symptoms of COVID-19 with concomitant pulmonary embolism creates a challenge for defining the diagnosis [3,20,21], and studies suggest that SPECT-CT combines the increased sensitivity of SPECT imaging with the high specificity of CT imaging [22] because the CT component of SPECT-CT is capable of exploiting the radiographic information present, making it useful for identifying the regions of the lung that are hypoventilated, and appears to be a viable alternative for the diagnosis of PE in individuals with COVID-19, although it is less effective than ventilation scintigraphy [4,6,15,22,23]. The included studies identified positive results for the diagnosis of PE using both CT perfusion scans [12,13,14,15,16,17,18,19] and CT ventilation [14,15,16], suggesting the efficiency of both methods. However, according to Voo and Dizdarevic 2020 [20], it is important to note that if ventilation scanning is considered to be indicated, it should only be performed if CTPA is contraindicated and in exceptional circumstances in confirmed or suspected cases of COVID-19.

Due to the persistent need to provide this critical diagnostic service without compromising staff and patient safety, two included studies [17,18], as well other studies from the literature during the COVID-19 pandemic [6,20,24], reported the use of perfusion-only SPECT-CT, without ventilation, as an efficient and definitive exam.

The included studies observed, in addition to PE, perfusion abnormalities, such as perfusion defects, defects associated with mosaic attenuation, defects due to pulmonary infiltrates from COVID pneumonia, a shunt pattern, venous thromboembolism, pulmonary fibrosis, bronchiectasis, emphysema, pleural effusion, pericardial effusion, mediastinal adenomegalies, cardiomegaly, GGOs, consolidations, pulmonary thrombosis, and mixed lesions (mismatch lesions, matched lesions). These results were common CT imaging features of patients with COVID-19 pneumonia [25,26].

Regarding SPECT exams, for ventilation, Bonnefoy et al. [14] and Roux et al. [15,16] used the radionuclides 99 mTc-Technegas^®^ and/or krypton gas (81 mKr), which is consistent with the study by Bahloul et al. [27], which used 99 mTc-labeled Technegas^®^. On the other hand, Evbuomwan et al. used the 20–25 mCi radionuclide 99 mTc-DTPA to perform the tests. Jögi et al. [28] suggest in their findings that Technegas^®^ is the preferred radioaerosol, when compared to DTPA, because the overall unevenness of radiotracer deposition and the degree of central deposition are more pronounced in (99m)Tc-DTPA than in Technegas^®^ studies. Therefore, because of the better peripheral penetration, the extent of reverse mismatch was less when Technegas^®^ was used; but these findings are particularly related to obstructive disease. Thus, although there was a difference between the radionuclides used, all the aerosols are specific and indicated to evaluate the ventilation in the SPECT scan [4,22,28]. However, when it comes to individuals with COVID-19, if possible, this type of exam should be avoided.

For perfusion scans, two studies [16,17] used 3–5 mCi of 99 mTc MAA as the radiopharmaceutical, whereas De et al. [13] used 4–6 mci of 99 mTc MAA and Bahloul et al. [27] also used 99 mTc-labeled macroaggregated albumin. In contrast, Zolotnitskayä and Titova (2021) [19] did not specify the radiopharmaceuticals used. However, according to the European Association of Nuclear Medicine (EANM) practice guidelines and the recent guidelines on V/Q SPECT-CT image exams, intravenously injected macroaggregates of 99 mTc MAA-marked human albumin with a diameter of 15–100 μm are almost universally used [4,22,24].

Evbuomwan et al. [16], Das et al. [18], Ozturk et al. [17], and De et al. [13] noted that D-dimer elevation is often seen in patients with COVID-19 due to the acute lung injury itself or due to thromboembolic complications, which can occur due to COVID-19; this was also observed in the review by Paliogiannis et al. [29] and in the study by Léonard-Lorant et al. [30]. However, not all published studies are concerned with carrying out this assessment despite knowing its importance in complementing the data from patients with COVID-19, since regular screening and D-dimer monitoring reflect the severity of the disease and guide anticoagulation therapy [31].

According to the results, a protocol for the detection, staging, and characterization of patients with positive COVID-19 utilizing alternative imaging methods would be interesting for use in clinical practice.

In a systematic review of the literature, Rahmanipour et al. (2023) pointed out that various perfusion patterns in COVID-19 are challenging but can be alleviated by adding SPECT/CT to lung perfusion exams. Moreover, it is highlighted that perfusion-only SPECT/CT can rule out or rule in other perfusion patterns in a relevant number of patients, and ventilation scans might be important for some patients [32].

Regarding the critical analysis of the risk of bias performed, although the included articles were generally of good methodological quality, the review exposed possible flaws that could compromise the internal validity of the studies.

The current systematic review has some limitations, and the results need to be interpreted with caution. Despite the positive results, the included studies used different types of SPECT scans, and a diversity of techniques, radiopharmaceuticals, protocols, and various pulmonary abnormalities were identified, which makes it challenging to interpret the findings and to draw convincing conclusions about which type of SPECT scan is ideal to perform on patients, regardless of the severity of the COVID-19 infection, and its risks and benefits. This takes into account that there are studies that use ventilation, for example, a technique that should be avoided according to the literature. Additionally, this type of SPECT/CT imaging examination has limitations in terms of accessibility, costs, and potential technical challenges in pandemic scenarios, mainly in terms of contamination.

A strength of the current study would be definitions of possible abnormalities and parenchymal lesions seen on different SPECT/CT scans in populations in different countries suffering from active COVID-19. Moreover, it demonstrates the relevance of the association of CT with SPECT, which allows the maximum extraction of benefits from the image and demonstrates the addition of important information in relation to the diagnosis, treatment, or prognosis of these patients.

## 5. Conclusions

In the age of precision medicine, SPECT/CT scans in patients with COVID-19 can address the clinical knowledge and management of SARS-CoV-2-induced lung abnormalities. This suggests efficiency in a differential diagnosis that includes respiratory diseases of different etiology, and, with diagnostics and additional analyses, makes it possible to define a suitable therapeutic strategy for each patient. Perfusion is the type of SPECT scan image that seems to be efficient, safe, and most commonly used in these patients. The possible pulmonary complication most observed post-COVID-19 is pulmonary embolism. In future, further studies, including randomized controlled clinical trials of good methodological quality, are necessary to help better understand the types of SPECT images most commonly used and the main pulmonary parenchymal lesions observed in the images of individuals with COVID-19.

## Figures and Tables

**Table 1:** Characteristics of the included studies in the review.

Author/Year	Country	Demographic Characteristics	Severity of COVID-19	SPECT Exam Type	Radiopharmaceutical	Lung Complications and Diagnoses (Pre- and Post-Exam)	Results	Study Design/LE
Evbuomwan et al. 2021 [16]	South Africa	n = 56 Male = 7 Female = 49 Age = 48 (41–54) years old	Non-hospitalized patients diagnosed with mild COVID-19 infection after de-isolation.	SPECT/CT and V/Q SPECT/CT	Ventilation studies: 20–25 mCi of 99 mTc-DTPA using the SmartVentTM (Diagnostic Imaging Limited, Elkington Lodge, UK) radioaerosol delivery system. Perfusion study: 3–5 mCi of 99 mTc MAA.	Perfusion defects, defects in keeping with PE, defects associated with mosaic attenuation but not due to pulmonary embolism, defects due to pulmonary infiltrates from COVID pneumonia, mosaic attenuation on CT, and showing a pattern likely consistent with shunting on the perfusion images.	Perfusion abnormalities in the lungs, such as PE, parenchymal infiltrates, or other causes of mosaic attenuation specific to the pathophysiology of COVID-19 infection, are frequently found in non-hospitalized patients with COVID-19 with mild disease. Highlights the significance of V/Q SPECT/CT imaging in identifying and distinguishing various types of perfusion abnormalities.	Retrospective cohort study/III-2
Das et al. 2021 [18]	USA	n = 6 Male = 3 Female = 3 Age = 31–70 years old	Patients hospitalized with SARS-CoV-2.	Q-SPECT/CT	No IV contrast was administered.	PE, pneumonia.	In patients with COVID-19, the presence of a contraindication for iodinated contrast media and a moderate or high pre-test probability for PE, Q-SPECT/CT has clinical utility for diagnosing PE.	Short communication—retrospective study/III-2
Sajal De et al. 2021 [13]	India	n = 54 Male = 44 Female = 10 Age = 55.4 (34–76) years old	n = 14 moderate COVID-19 infections. n = 40 severe COVID-19 infections.	SPECT/CT	4–6 mCi of 99 mTc-MAA.	Venous thromboembolism and PE.	Mismatched perfusion defects suggestive of pulmonary thromboembolism were observed despite prophylactic anticoagulation. A poor predictor of mismatched perfusion defects is the serum D-dimer level in patients early after COVID-19. Imaging studies confirmed evidence of PE, which should support the decision to extend anticoagulant prophylaxis in patients post-COVID-19.	A single-center retrospective study/III-2
Ozturk et al. 2021 [17]	Turkey	n = 77 Male = 29 Female = 48 Age = 53.9 ± 13.6 years old	mild-to-moderate course of COVID-19.	Q-SPECT/CT	3–5 mCi Techhnetium 99m-labeled macroaggregated albumin (Tc-99m MAA).	PE	During the post-COVID phase, Q-SPECT/CT serves as a highly effective primary choice image technique for detecting pulmonary microembolism. Even patients with mild-to-moderate COVID-19 who are not hospitalized exhibit a tendency toward thrombosis. Therefore, anticoagulant prophylaxis should also be considered for these individuals throughout the course of the illness.	Retrospective, cross-sectional/III-2
Zolotnitskayä & Titova 2021 [19]	Russia	n = 136 Male = 66 Female = 70 Age = 56.0 ± 17.4 years old	n= 59 patients on an outpatient basis, n= 77 from a hospital. All patients had a COVID-19 infection at different times from the onset of the disease.	SPECT/CT	Radiopharmaceutical not specified at a dose of 1–1.5 mBq per 1 kg of body weight.	Interstitial lung diseases (pulmonary fibrosis, bronchiectasis).	The scan images showed alterations in microcirculation, regardless of the severity of the condition. The development of fibrotic changes, leading to the progression of interstitial lung disease linked to the virus, may suggest a gradual reduction in microcirculation in the lower lung regions. This could result in localized areas of hypoperfusion with critically low radiopharmaceutical uptake, persistent lung tissue densification resembling a “ground-glass” pattern, reticular alterations, the emergence of traction bronchiectasis, reduced lung diffusion capacity, and a decline in alveolar volume.	No information/III-2
Bonnefoy et al. 2022 [14]	France	n = 145 Male = 67 Female = 78 Age = ±71 years old	Patients with confirmed COVID-19 infection.	V/Q SPECT/CT and SPECT/CT	Perfusion: 99 mTc-labeled human albumin (99 mTc-MAA). Ventilation: 99 mTc-Technegas^®^ or krypton gas (81 mKr).	Chronic lung lesions (i.e., emphysema and/or fibrosis lesions), pleural effusion, pericardial effusion, mediastinal adenomegalies, cardiomegaly, PE, GGOs, consolidations.	A large proportion of patients with COVID-19 with suspected acute PE are assessed with V/Q SPECT/CT imaging to show parenchymal lung lesions on CT, with a frequent impact on ventilation and/or perfusion. COVID-19-related pulmonary lesions, considering in order of frequency and functional impairment, consolidation, CP, and GGO, with typically a reverse mismatched or a matched pattern. However, supporting the potential interest of combining the functional and morphological approach with V/Q SPECT/CT imaging for the assessment of patients with COVID-19, with a wide heterogeneity in the severity of lung ventilation alteration and perfusion.	A retrospective central analysis/III-2
Farkas et al. 2022 [12]	Hungary	n = 91 Male = 46 Female = 45 Age = 71.4 ± 13.9 years old Smoking = 5	Positive diagnosis of SARS-CoV-2 virus infection and clinical symptoms suspicious of PE.	Q-SPECT/CT	200 MBq (5.4 mCi) of Tc-99m macroaggregated albumin (MAA—Macro-Albumon, Medi-radiopharma Ltd., Érd, Hungary).	Moderately severe parenchymal lesions, such as GGOs, consolidations, and mixed lesions (mismatch lesions, matched lesions, reverse mismatch), PE, pulmonary thrombosis.	Most patients with COVID-19 exhibited heterogeneous perfusion abnormalities, including parenchymal lesions with normal, increased, or decreased perfusion as well as perfusion defects in otherwise healthy lung regions. These findings may be attributed to the dysfunction of the hypoxic pulmonary vasoconstriction mechanism and the presence of pulmonary thrombosis and embolism.	A single-center, retrospective study/III-2
Le Roux et al. 2022 [15]	France	n = 183 patients, Male = 66 Female = 117 (64%) Age: 74 (15–102) years old. >80 years old = 68 (37%) >90 years old = 26 (14%)	Patients with COVID-19 with suspected PE.	perfusion SPECT, perfusion SPECT/CT, and V/Q SPECT/CT	Ventilation using Technegas^®^ (Cyclomedica Australia Pty.Ltd, Kingsgrove, New South Wales) aerosol and 81 mKr gas.	PE	In only 57% of patients with COVID-19 assessed with lung scintigraphy, PE could confidently be excluded without ventilation imaging. In 31% of patients, ventilation imaging was required to confidently rule out PE. Overall, the prevalence of PE was 12%, considered low.	A multicenter study/III-2

MAA: macroaggregated albumin; GGO: ground-glass opacities; PE: pulmonary embolism; CT: computed tomography; SPECT/CT: single-photon emission computed tomography; V/Q SPECT/CT: ventilation/perfusion single-photon emission computed tomography; Q-SPECT/CT: perfusion single-photon emission computed tomography; SARS-CoV-2: severe acute respiratory syndrome coronavirus 2; COVID-19: coronavirus disease 2019.
